# Supplementary material for: Accelerated Aging Characterizes the Early Stage of Alzheimer’s Disease
Source: Cells. 2022 Jan 11;11(2):238. doi: 10.3390/cells11020238 (PMC8774248; doi:10.3390/cells11020238)
Supplement: Supplementary file 1 [file cells-11-00238-s001.zip › cells-1426539-supplementary.pdf]

SUPPLEMENTARY MATERIALS

Animal Preparation and Surgery

Animal preparation for surgery and recordings was performed as described in Leparulo et al., 2019 [1]. Mice were anesthetized by intraperitoneal injection of urethane (1.5 g/kg, Sigma–Aldrich, Milan, Italy) dissolved in 0.9% NaCl, and a mixture of xylazine/tiletamine-zolazepam (XTZ) (Rompun 1 mg/Kg plus Zoletil 10 mg/kg) dissolved in phosphate buffer. Anesthesia induction was done by an initial dose of urethane followed, 30 min later, by a single dose of XTZ. The absence of reaction to noxious stimuli (e.g., tail, hind paw and ear pinches) ensured a constant level of anesthesia. Coupling XTZ with urethane anesthesia substantially reduced the mouse death rate from 50 to 20%. After shaving the fur over the head, mice were restrained in a stereotaxic frame and the skull was exposed. A hole was drilled on the skull over the left hemisphere at the site for insertion of the multi-site recording probe. To locate the dentate gyrus (DG) for probe insertion, we used landmarks on the skull of each mouse, according to the procedure described [2] (~2.2 mm posterior to bregma, ~1.1 mm lateral to midline). The cavity over the skull was filled with Krebs-Ringer solution and an external Ag-Cl reference electrode was dipped within. Body temperature was kept at 37 ± 0.5 °C all the time, using a servo-controlled heating pad (ATC1000-World Precision Instruments, Inc., Friedberg, Germany). At the end of the electrophysiological experiment, the mouse was euthanized by excess of anesthesia and the brain was dissected.

Signal Acquisition

Spontaneous LFP activity was acquired by a linear 32-electrode-silicon probe (ATLAS Neuroscientific, Probe: E32-100-S1-L6-NT; pointy tip feature; 100 μm spaced electrodes; mean impedance 0.28 MΩ in standard Krebs' solution) connected by a 32-channel head stage (Intant, RHD2000; 30 kHz sample rate, Lowpass 0.1 - 500 Hz, Highpass 100 Hz - 20 kHz, 2.4 μV RMS noise, 16bit resolution) and an SPI cable to the acquisition system (Open Ephys, OEps Tech, Lisbon, Portugal, v.0.4). The probe was inserted into the posterior parietal cortex (PPC) and lowered up to 2.3 mm, across the hippocampal formation (HPF), so that the first 24 channels of the probe were recording from the deepest layer of DG up to the cortical layers. The LFP signals were visualized, recorded, and digitalized at 10 kHz through the Open Ephys acquisition software. Together with the LFP signals, other physiological signals were simultaneously recorded to determine health conditions. Heartbeat was monitored through electrocardiogram (ECG) recording, 10X amplified and filtered between 1 and 100 Hz by means of a DAM 50 Amplifier (World Precision Instruments, Friedberg, Germany). ECG positive and negative derivations were subcutaneously inserted into the forelimbs. Respiration-induced movements of the chest wall were converted in voltage fluctuations by the piezoelectric properties of the temperature probe (IT-23, World Precision Instruments, Friedberg, Germany). The respiration signal was amplified 100X and band-passed between 0.1 and 100 Hz by means of a DP-301 amplifier (Warner Instruments, Crisel, Rome, Italy). Physiological signals were digitalized at 10 kHz by a PCI-6071E I/O card (−0.5 to 0.5 V input range) combined with a BNC-2090 terminal block (National Instruments, Rome, Italy) in differential mode and recorded through a custom-made LabView (National Instruments, Rome, Italy) script. Upon reaching a stable level of anesthesia (see below for quantitative evaluation of anesthesia level), we recorded the spontaneous brain activity for 30 minutes.

Data Processing and Analysis

For quantitative comparisons we chose seven channels located at the following depths: (i) 2100 and 1900 μm, in the lower and upper stratum granulare (*lo-sg* and *up-sg*) of DG; (ii) 1500 and 1200 μm, in stratum radiatum-lacunosum molecolare (*sr-lm*) and stratum pyramidale (*sp*) of CA1; (iii) 900, 600, and 300 μm in Layer 6 (L6), L4/5 and L2/3 of the posterior parietal cortex (PPC). We also monitored the mean heart and respiration rates to check the health status of the mouse to select the proper windows for data analyses.

Data analysis of electrophysiological signals was performed offline in Matlab (v.2018b, Mathworks Inc., Natick, MA, USA) using custom-written scripts. Firstly, the individual data files containing the acquired

signals from different channels were converted from Open-Ephys format to Matlab file format. The analysis of the acquired raw data consisted of a pre-processing step, in which raw signals were cleaned through the application of a Gaussian filter to remove 50 Hz noise and its harmonics. The signals from the first 24 channels were filtered through the built-in non-causal zero-phase distortion filtering function (*filtfilt.m*), which processes the data in both forward and reverse directions to avoid any phase distortion, with coefficients from the built-in Butterworth transfer function (*butter.m*). Baseline drift was removed from ECG and respiration signals using a median estimation method. Secondly, the signals were low-pass filtered (filter order: 5; cut-off frequency: 190 Hz for LFP, 25 Hz for ECG, and 10 Hz for respiration) and down-sampled (using the *downsample.m* function) to 500 Hz, 50 Hz and 20 Hz, respectively. Finally, in the LFP recordings, windows were selected for the analyses based on the stability of heart and respiration rates as previously described [1]. In the final step, time-locked LFP signal windows corresponding to the overlapping stable signal portions were extracted and a 5-min window was selected for all the analyses. This pre-processing step was necessary to ensure that all the animals were stable during the acquisition procedure.

Power and Cross-Frequency-Coupling analyses

LFP power spectral density (PSD) was calculated for a 2.5-min window through the Welch estimate method (*pwelch.m*) as described [3]. Hamming windows of 2 s were used with an overlap of 50%. The spectral power in the different frequency bands was obtained by computing the integral with the trapezoidal method (*trapz.m*) under the PSD curve, with frequency resolution of 0.01 Hz. For the analysis of the Cross-Frequency-Coupling (CFC) index of SO (or delta) with higher bands, a 5-min window was firstly Butterworth-filtered in the SO and delta bands (3rd order filter) and then in the specific higher frequency band (6th order filter). Next, the Hilbert transform (*hilbert.m*) was applied to each time-series obtained from filtering. Then phase, angle and amplitude time-series were extracted from the Hilbert transform of SO and delta and the higher frequency filtering, respectively. Finally, PAC index was computed by means of the general linear model (GLM) as described in [4]. The GLM method was chosen owing to its sensitivity [5]. Basically, the amplitude of the faster oscillation is modelled by multiple regression and the index is the proportion of variance explained by the model.

Cross-correlation of instantaneous maximal LFP amplitudes

For the cross-correlation analysis we implemented previous methods [6]. The recording windows used for PAC analysis were band-pass filtered for the SO and delta bands, and the instantaneous amplitude of each signal was obtained from the Hilbert transform by a custom-written script. Subsequently, for all channels, maximal amplitude cross-correlation matrices were built with the Matlab function xcorr (*xcorr.m*) over latencies ranging from + 0.1 to − 0.1 s. The mean amplitude was first subtracted from each vector prior to cross-correlating, as the DC component of a signal has no relevance for cross-correlation. The latency (lag) at which the cross-correlation peaked was then determined. Matrices of maximal cross-correlation coefficients and associated lags were obtained for each mouse and then averaged by genotype and age-cohort.

UP/DOWN-state analysis, spike and burst detection

Spiking signals were extracted by band-pass filtering pre-processed data at 0.3-3 kHz using a zero-lag Butterworth filter of order 3. UP- and DOWN-states were detected starting from LFP traces (Supp. Fig. 9A) performing the following steps: i) computation of the standard deviation (i.e., SD) of the LFP traces within sliding windows of 4 samples in order to accentuate the signal variations (Supp. Fig. 9B); ii) smoothing of the SD signals using a moving-average filter (Supp. Fig. 9C); iii) thresholding of the previously smoothed signals using the mean value as threshold (red line in Supp. Fig. 9C); iv) classification of UP- and DOWN-states (i.e., temporal epochs above threshold were defined as UP-state, while temporal epochs below threshold as DOWN-state), as shown in Supp. Fig. 9D; v) merging of UP-states occurring less than half a second from each other, as shown in Supp. Fig. 9C where two detected UP-states (highlighted in green) are merged being spaced by 200 ms (Supp. Fig. 9D). Afterwards, we estimated UP- and DOWN-state duration. In particular, the time between the end of an UP-state and the start of the following one corresponds to a DOWN-state duration,

while the time between the end of a DOWN-state and the start of the following one corresponds to an UP-state duration.

Spike detection was performed on the spiking traces using WaveClus software [7], an unsupervised and fast method for spike detection. Specifically, spikes were detected using an automatic amplitude threshold set as a multiple (3.5x) of an estimate of the standard deviation of the background noise. Spike detection allowed to compute the Mean Firing Rate (MFR) from the total number of spikes detected within the 5-min acquisition time. Burst detection was performed by adopting a method based on Inter-Spike Interval (ISI) duration, i.e., the time between consecutive spike pairs, with a criterion for a minimal number of spikes with short ISIs [8]. To this aim, burst detection was performed by computing the ISI and by selecting only those spikes close to each other less than 100 ms. In addition, a burst was defined as a set of at least three spikes. Similarly, burst detection allowed to compute the Mean Bursting Rate (MBR) from the total number of bursts detected within the 5-min acquisition time. Subsequently, spike and burst detection was performed within each previously detected UP-state in order to combine information carried by both low and high frequency activity.

Histology and immunohistochemistry

Upon brain dissection, left hemispheres were quickly dissected, post-fixed in 4% PFA for 48 h at 4°C. After washing 3 times with phosphate buffered saline (PBS), hemibrains were cut parallel to the coronal or sagittal plane with a vibratome (Leica VT1000 S), yielding 70-μm-thick slices. Brain slices were cryopreserved in 50% PBS, 30% ethylene glycol, and 20% glycerol at -20 °C until their use. Immunostaining was performed on floating slices. Slices were incubated in permeabilizing/blocking buffer containing 0.5% TritonX-100 and 5% normal donkey serum in phosphate-buffered saline (PBS) for 1 h at room temperature (RT). Next, they were first incubated overnight at 4°C with rabbit anti-GFAP (Dako, 1:500), mouse anti-Aβ (McSA1, Medimabs, 1:500), mouse anti-APP/Aβ (4G8, Biolegend, 1:500), goat anti-Iba1 (Abcam, 1:1000), rabbit anti-Lamp1 (Abcam, 1:500) and then for 2 h at RT in the dark, with the secondary antibodies Alexa488-conjugated donkey anti-rabbit (Invitrogen, 1:500), Alexa555-conjugated donkey anti-mouse (Invitrogen, 1:500), or Alexa647-conjugated donkey anti-goat (Invitrogen, 1:500). Slices were mounted on microscope slides by means of Aquapolymount (Polysciences), and stored at 4°C until visualization. Each step described above was separated from the next one by 3 washes in PBS (10 min each).

Confocal imaging and image analysis

Slices were imaged with a Leica TCS SP5 II, upright confocal microscope equipped with an HCX PL Fluotar 20×/0.50 air or an HCX PL APO 100×/1.40 oil objective. Confocal images were acquired at 1024×1024 pixels per image, by averaging 2 frames at 100-Hz-acquisition rate. During acquisition, parameters were kept constant, with equal exposure times per channel and with the same graphical pre-processing within one experiment. For analysis and quantification, the ImageJ (NIH, USA) software was used. Images are representative of 3 animals per age cohort.

References

1. Leparulo, A.; Mahmud, M.; Scremin, E.; Pozzan, T.; Vassanelli, S.; Fasolato, C. Dampened Slow Oscillation Connectivity Anticipates Amyloid Deposition in the PS2APP Mouse Model of Alzheimer's Disease. *Cells* **2019**, *9*, 54.
2. Huang, Y.; Yang, S.; Hu, Z.Y.; Liu, G.; Zhou, W.X.; Zhang, Y.X. A new approach to location of the dentate gyrus and perforant path in rats/mice by landmarks on the skull. *Acta Neurobiol Exp* **2012**, *72*, 468–472.
3. Fontana, R.; Agostini, M.; Murana, E.; Mahmud, M.; Scremin, E.; Rubega, M.; Sparacino, G.; Vassanelli, S.; Fasolato, C. Early hippocampal hyperexcitability in PS2APP mice: role of mutant PS2 and APP. *Neurobiol. Aging* **2017**, *50*, 64–76.

4. Penny, W.D.; Duzel, E.; Miller, K.J.; Ojemann, J.G. Testing for nested oscillation. *J. Neurosci. Methods* **2008**, *174*, 50–61.

5. Rubega, M.; Fontana, R.; Vassanelli, S.; Sparacino, G. A tunable local field potentials computer simulator to assess minimal requirements for phase–amplitude cross-frequency-coupling estimation. *Netw. Comput. Neural Syst.* **2016**, *27*, 268–288.

6. Adhikari, A.; Sigurdsson, T.; Topiwala, M.A.; Gordon, J.A. Cross-correlation of instantaneous amplitudes of field potential oscillations: A straightforward method to estimate the directionality and lag between brain areas. *J. Neurosci. Methods* **2010**, *191*, 191–200.

7. Quiroga, R.Q.; Nadasdy, Z.; Ben-Shaul, Y. Unsupervised Spike Detection and Sorting with Wavelets and Superparamagnetic Clustering. *Neural Comput.* **2004**, *16*, 1661–1687.

8. Grace, A.; Bunney, B. The control of firing pattern in nigral dopamine neurons: burst firing. *J. Neurosci.* **1984**, *4*, 2877–2890.

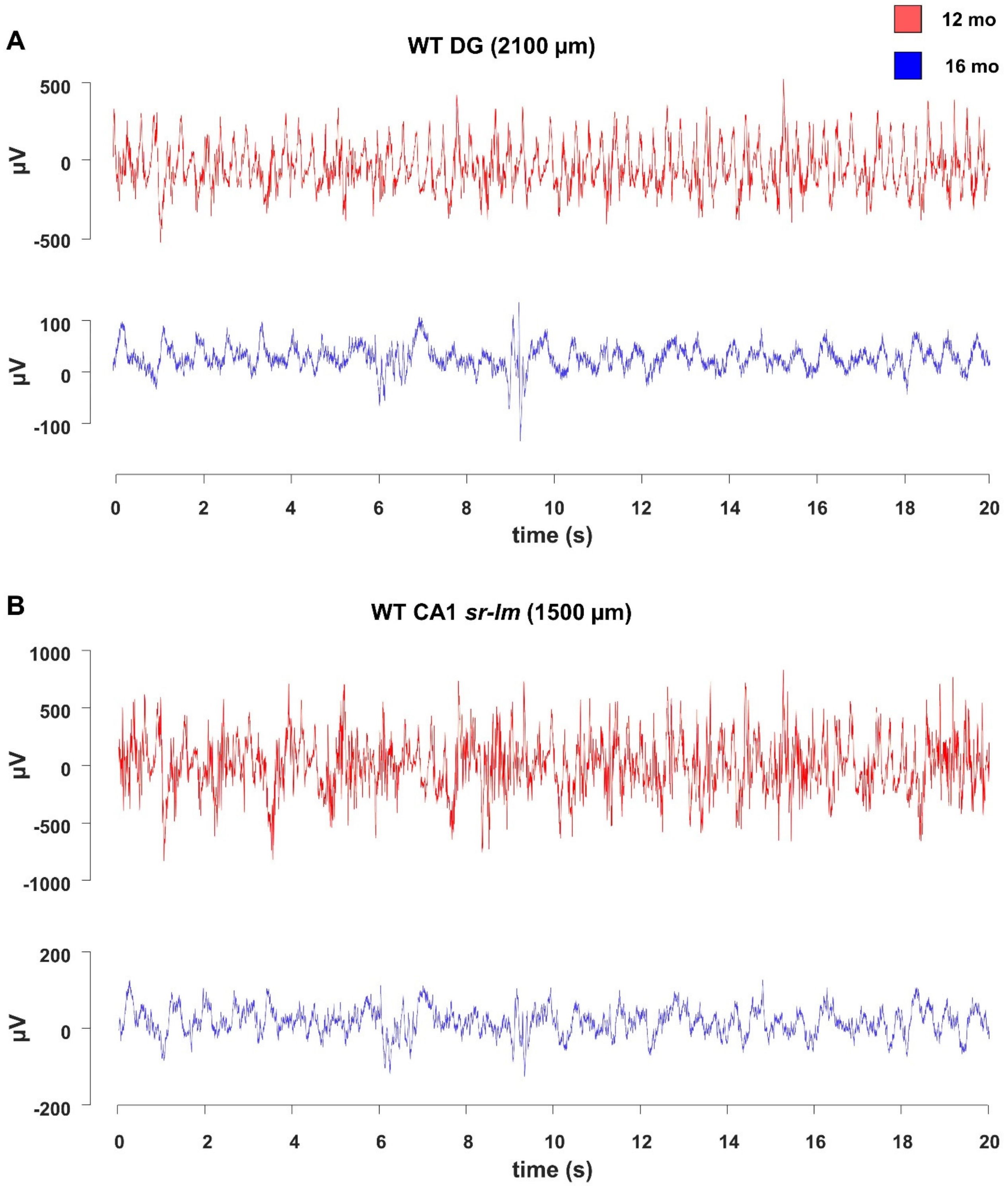

Supplementary Figure S1. Hippocampal activity in aged WT mice. Representative raw LFP traces in the DG (2100 µm) (A) and CA1 (*sr-lm*, 1500 µm) (B) of 12- and 16-month-old WT mice showing how activity is preserved despite the loss of total power in old mice.

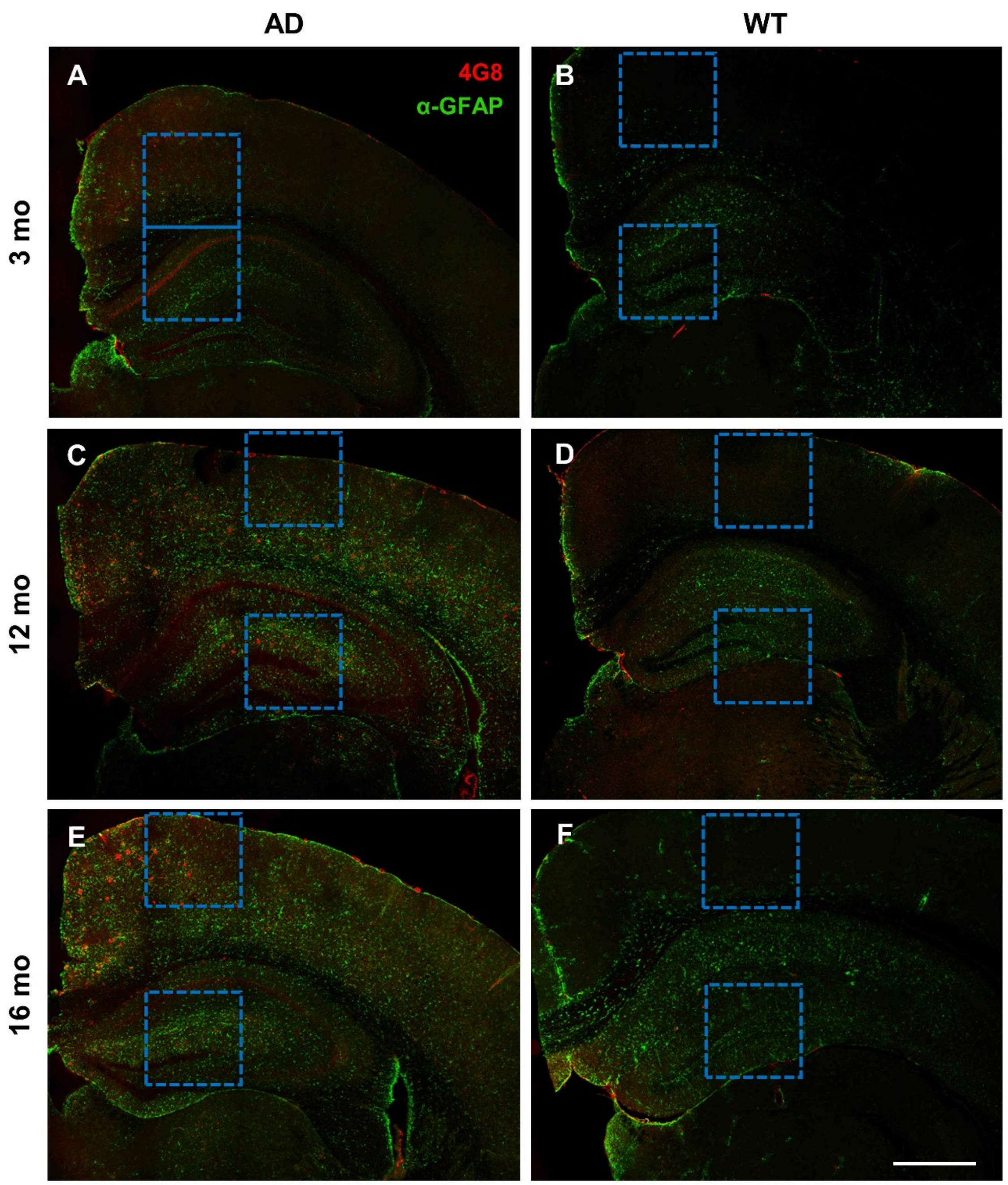

Supplementary Figure S2. High levels of Aβ plaque and gliosis in middle-aged and old AD mice. Confocal images of the coronal slices used for cropping the cortical and hippocampal images shown in Figure 1. Aβ and astrogliosis were detected with 4G8 (red) and anti-GFAP (green) antibody, respectively, in 3-, 12- and 16-month-old AD and WT mice (20x, scale bar 775 µm). Images are representative of 3 mice per genotype/age cohort.

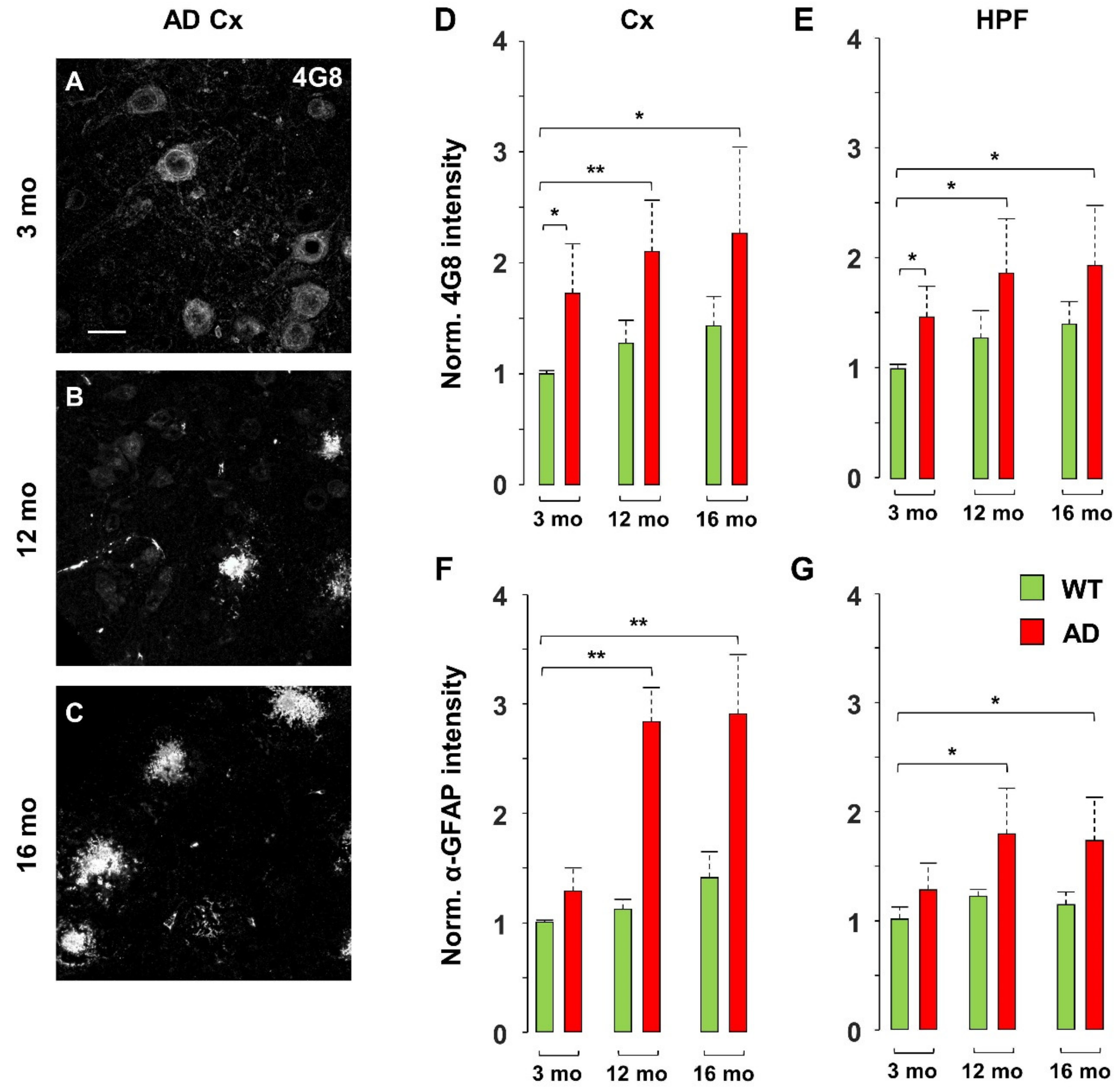

Supplementary Figure S3. Amyloidosis and gliosis in AD mice across ages. (A-C) Representative images of intracellular and extracellular 4G8 staining in cortical (Cx) slices from AD mice across ages (100x, scale bar, 20 µm). Quantification of 4G8 (D, E) and anti-GFAP (F, G) staining of cortex (Cx) and hippocampus (HPF). Values are the average intensity of 6 regions/mice normalized to the value measured in 3-month-old WT mice (mean + SEM, n = 3 mice) ** $p < 0.01$, * $p < 0.05$, Student's *t*-test).

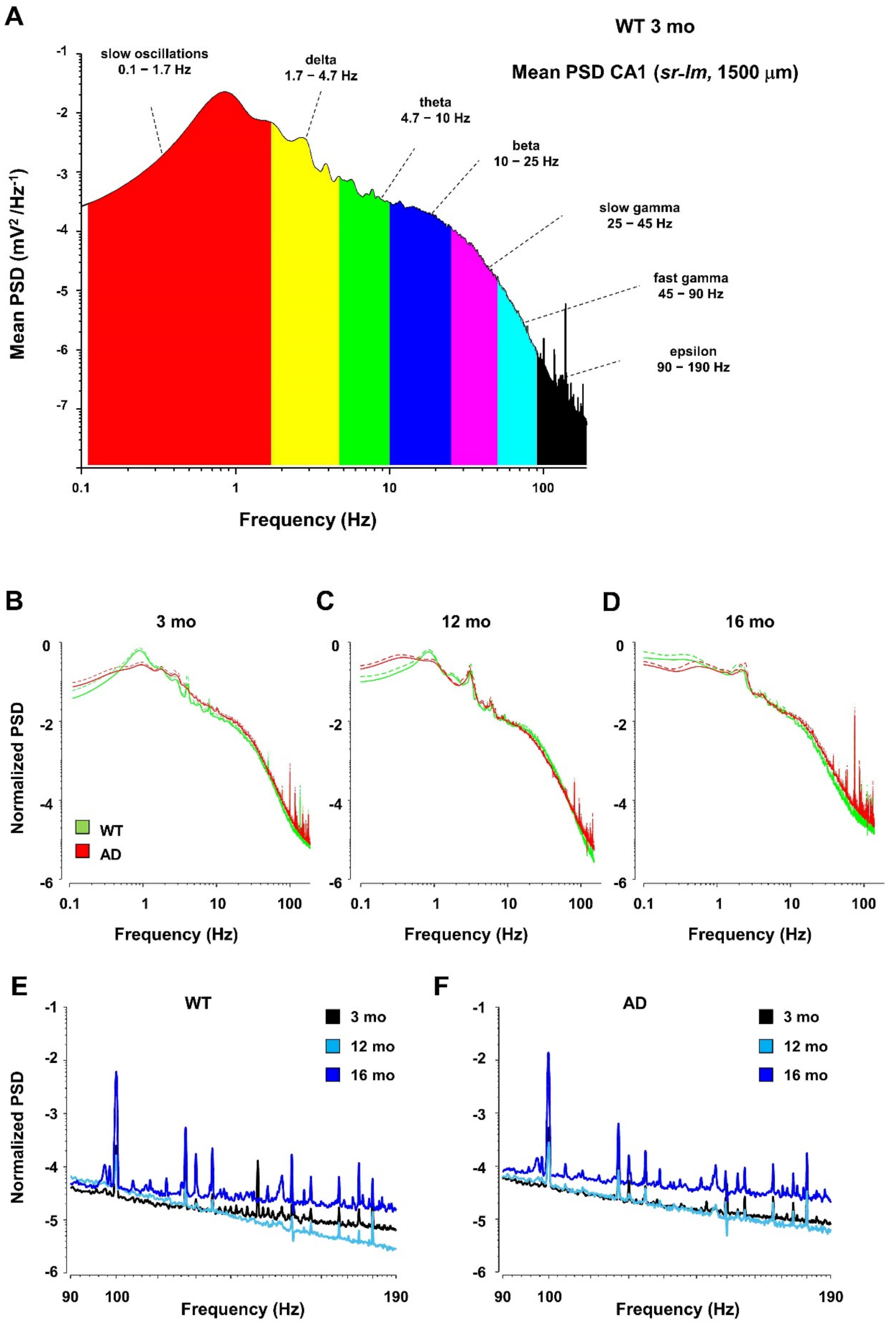

Supplementary Figure S4. Power Spectral Density plots for WT and AD mice across ages. (A) Representative mean PSD plot obtained from recordings in CA1 (*sr-lm*,1500 μm) in 3-month-old WT mice, also showing the frequency ranges used in this work for power analysis. For comparison of relative power changes, the PSD function was normalized to the total power measured at the same depth for each mouse, averaged and plotted (mean + SEM) in a log-log scale for WT and AD mice at 3 (B), 12 (C) and 16 (D) months of age. (E, F) Normalized PSD plots (mean + SEM) for WT and AD mice at 3 (black), 12 (red) and 16 (blue) months, overlaid by age in a log-semilog scale (90-190 Hz).

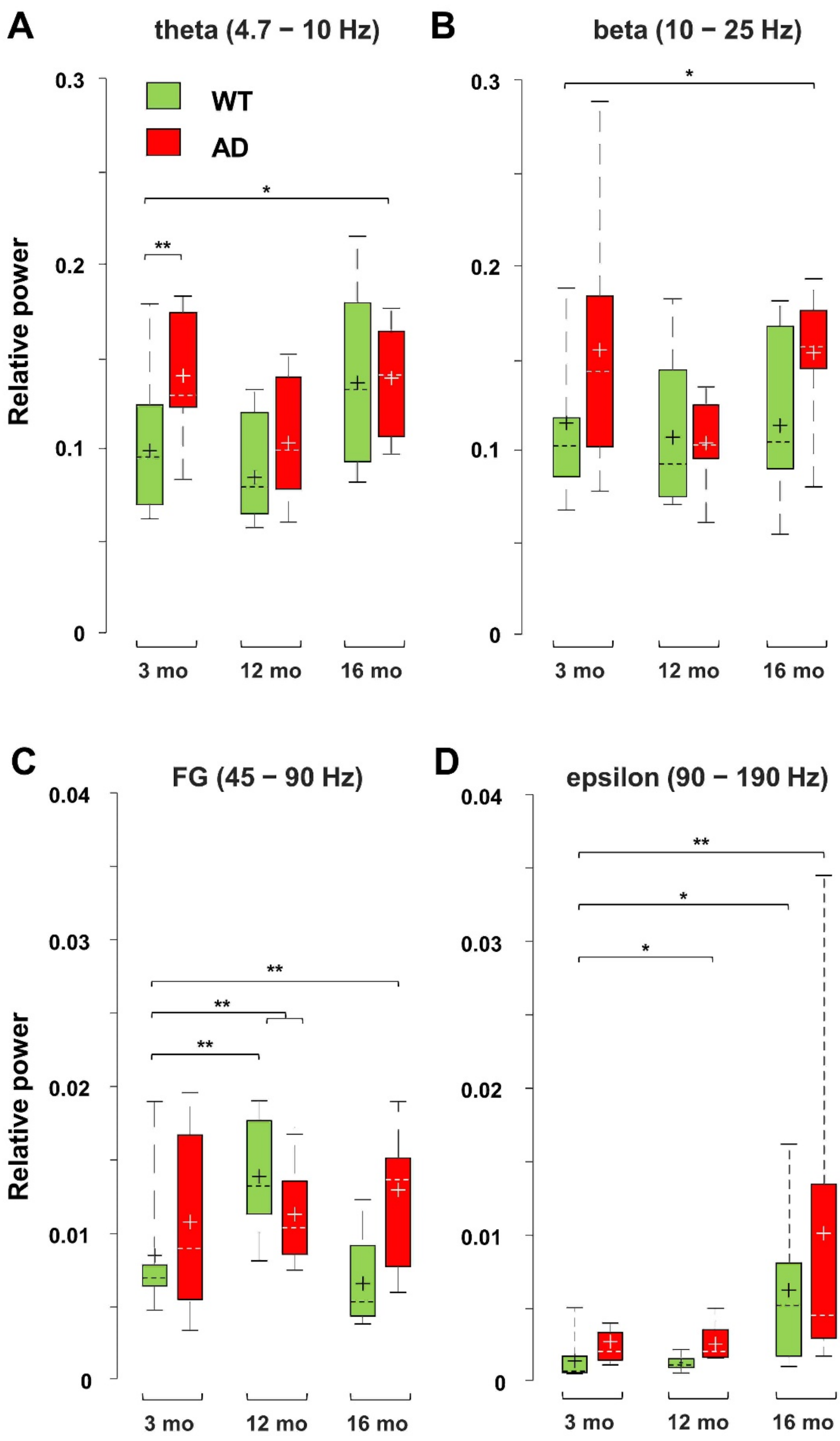

Supplementary Figure S5. Changes in the relative power of the High frequency bands in WT and AD mice across ages. Boxplots of the relative power of theta (4.7–10 Hz) (A), beta (10–25 Hz) (B), FG (45–90 Hz) (C) and epsilon (90–190 Hz) (D) bands in CA1 (*sr-lm* ,1500 µm) of WT and AD mice. At each frequency band, relative power is the percentage of total power at the indicated depth; * $p < 0.05$; ** $p < 0.01$.

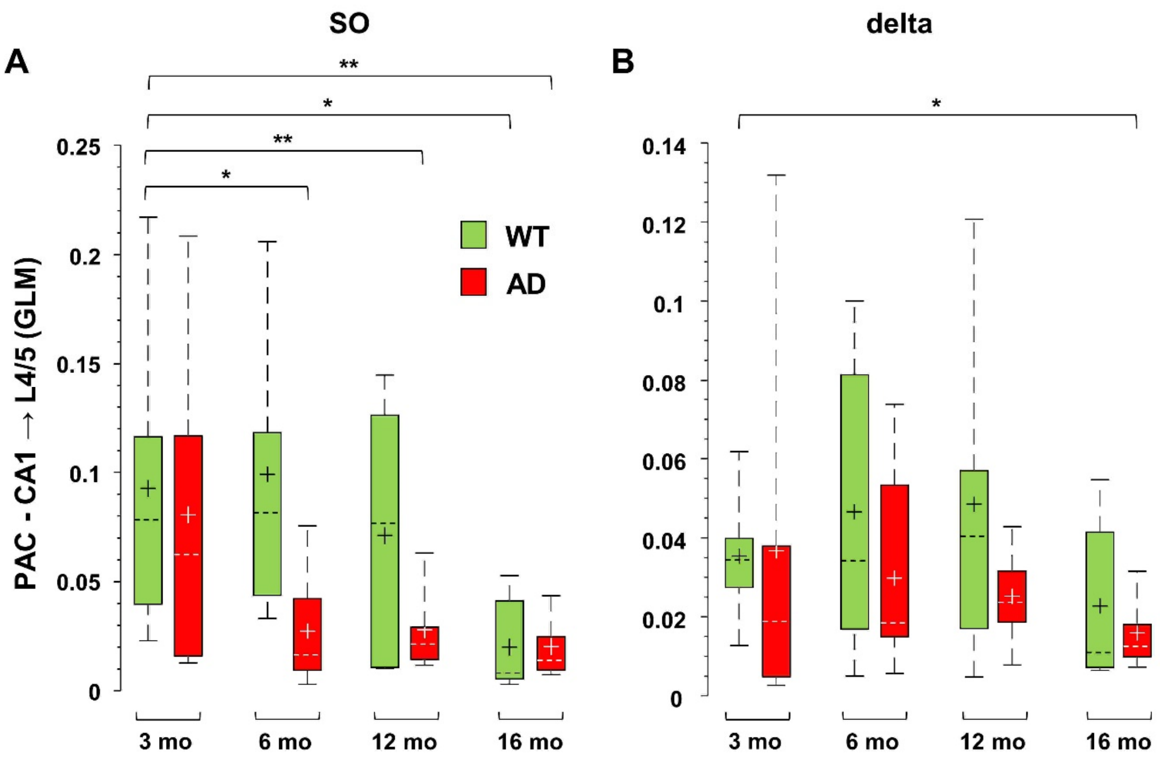

Supplementary Figure S6. Defective PAC between SO and epsilon band in AD and WT mice. PAC, namely the coupling between amplitude of low frequencies and phase of higher frequencies, is measured by the GLM index, as described in Supplementary Materials. (A) The PAC occurring between SO in CA1 (*sr-lm*, 1500 μm) and the epsilon band in L4/5 (600 μm) of the PPC is significantly reduced in old WT and AD mice, in the latter starting from 6 months. (B) The PAC between delta waves and epsilon band is reduced only in old AD mice, * $p < 0.05$; ** $p < 0.01$.

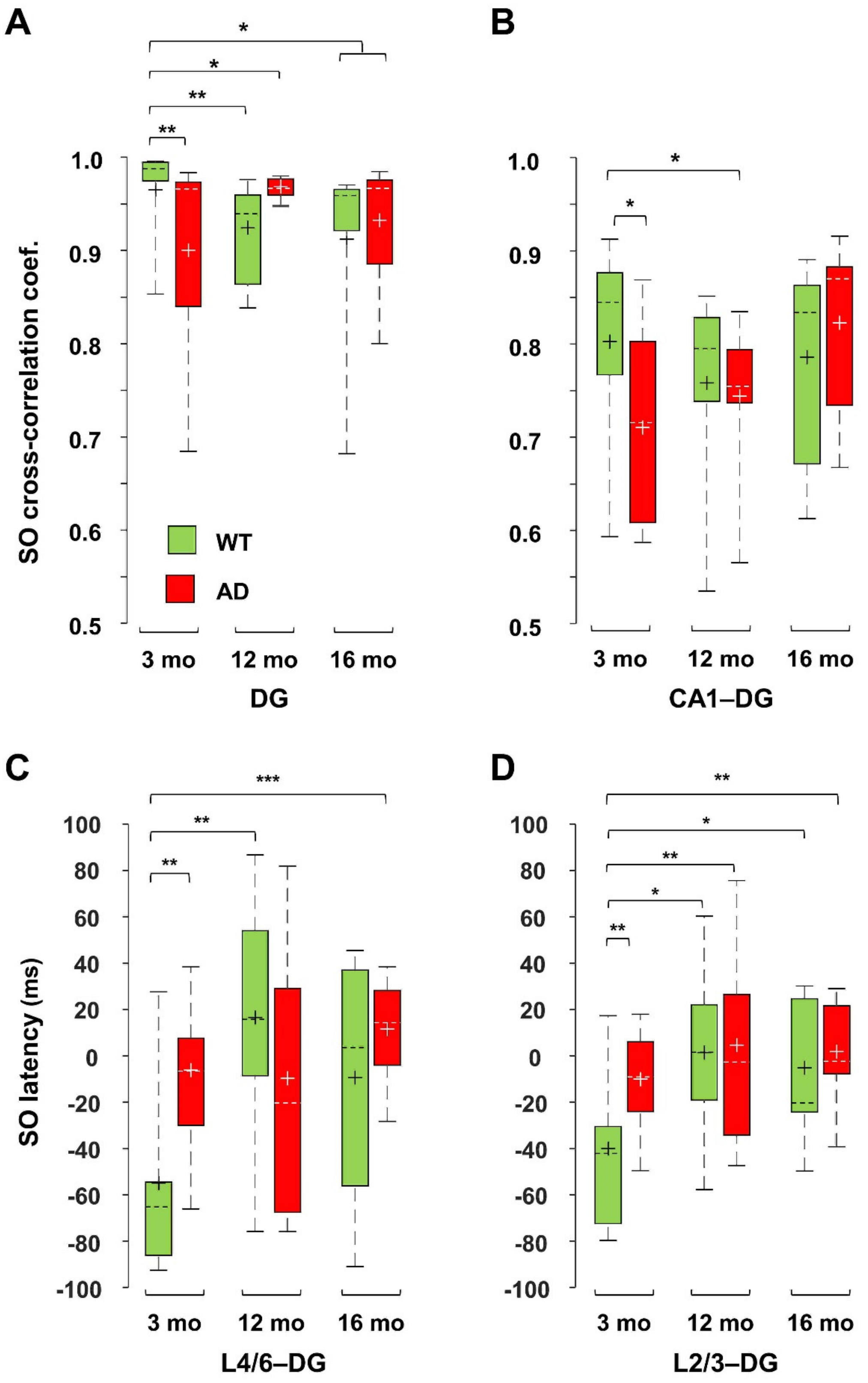

Supplementary Figure S7. Loss of SO cortico-hippocampal cross-correlation and latency occurs in aging WT mice and is anticipated in AD mice. For quantitative analyses of regional changes, SO maximal cross-correlation coefficients and latencies of each mouse were averaged within (intraregional, A) and between (cross-regional, B-D) regions, according to the scheme shown in Figure 6A. Values were then averaged by age and genotype and shown in boxplots as maximal cross-correlation coefficients (A, B) and latencies (C, D), * $p < 0.05$; ** $p < 0.01$; *** $p < 0.001$.

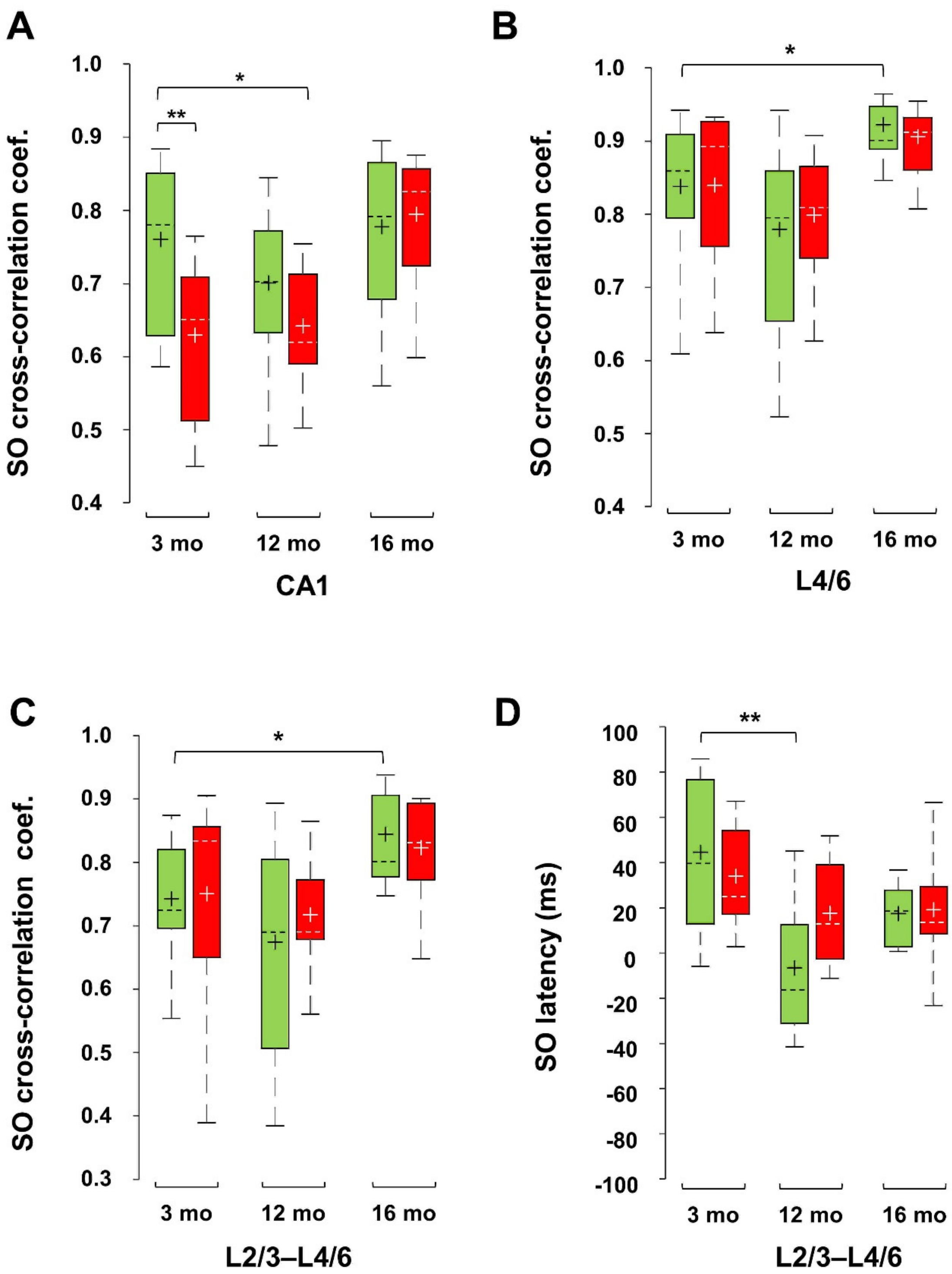

Supplementary Figure S8. Changes in SO cortico-hippocampal cross-correlation coefficients and latencies between AD and WT mice across ages. Analysis was carried out as shown in Figure 6A. Panels show the significant changes in cross-correlation or latency that occur in either AD (A) or WT (B-D) mice * $p < 0.05$; ** $p < 0.01$.

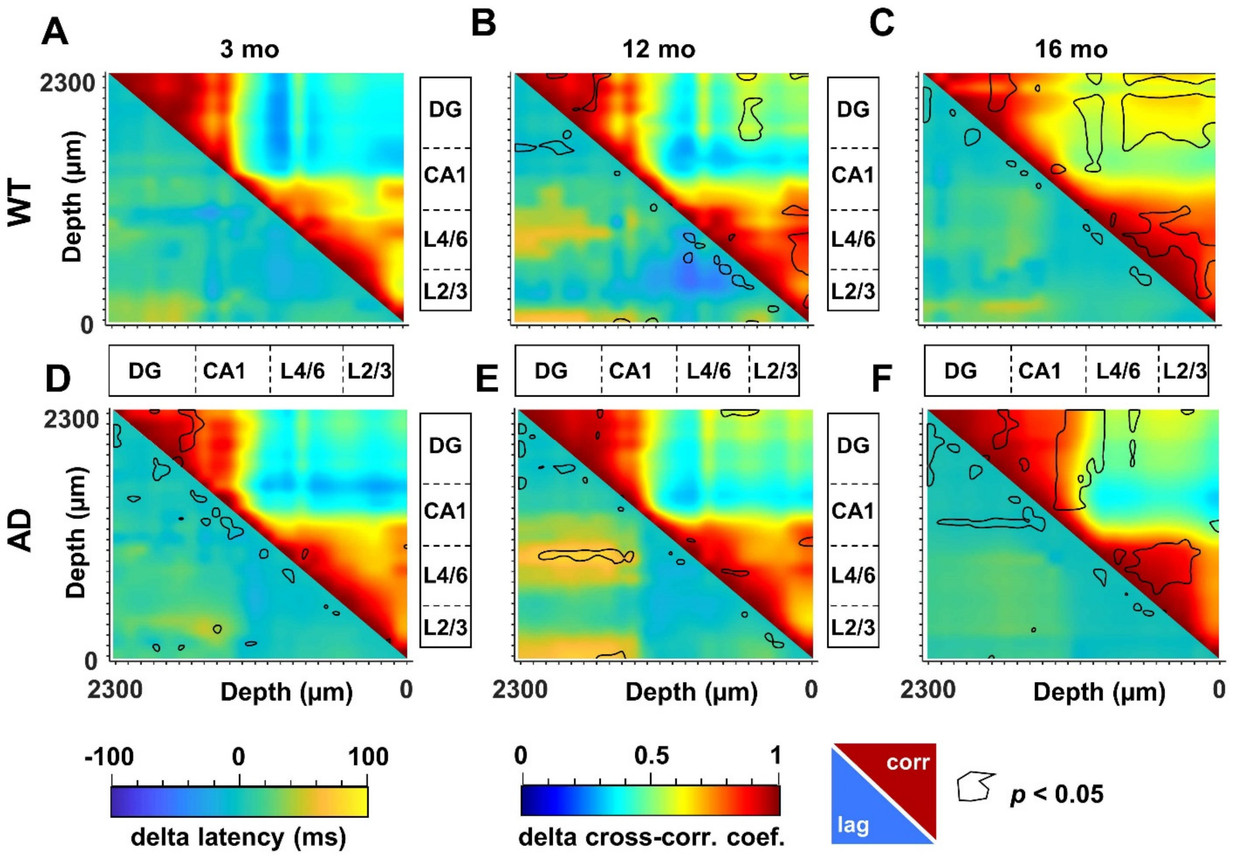

Supplementary Figure S9. Alterations in delta cortico-hippocampal connectivity occur in aged AD and WT mice. Delta connectivity was measured in terms of maximal cross-correlation coefficient and latency (lag) of the instantaneous delta amplitude, as described in Supplementary Materials. Matrices of cross-correlation coefficients (upper-right) and latencies (lower-left) for 3-, 12- and 16-month-old WT (A-C) and AD (D-F) mice were obtained by comparing each recording channel with all the other channels. The matrices report the areas with significant changes with respect to 3-month-old WT mice ($p < 0.05$, black line).

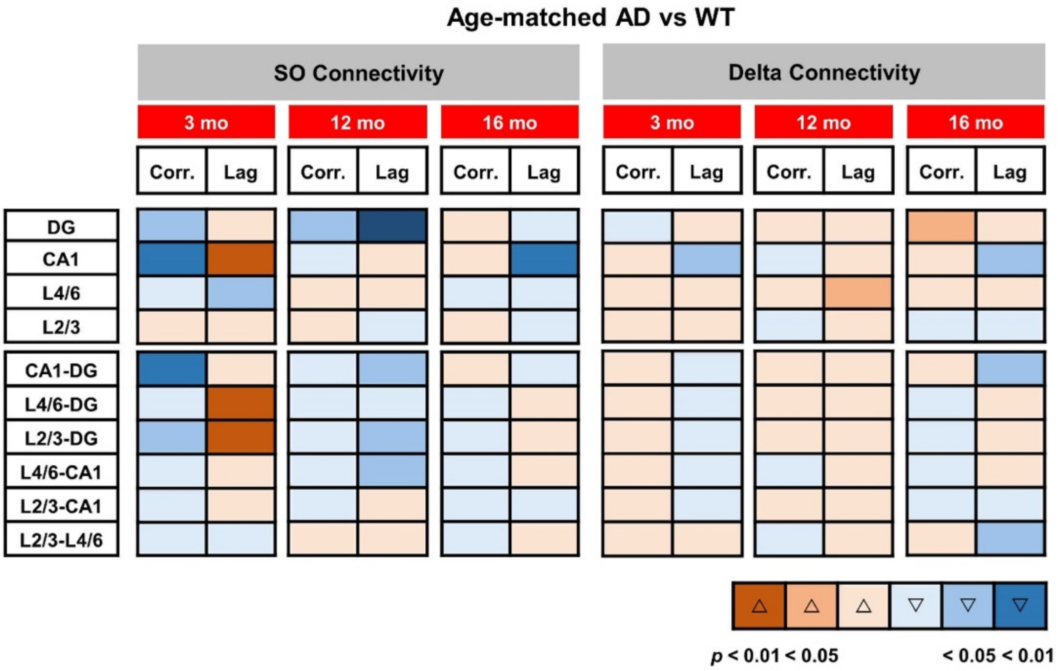

Supplementary Figure S10. Comparison of SO and delta connectivity by age-matching. For quantitative analyses of regional changes, maximal cross-correlation coefficients and latencies of each mouse were averaged within (intraregional) and between (cross-regional) regions, according to the scheme overlaid to the matrices of SO (A) and delta (C) connectivity shown in Figure 6. Synoptic views of the regional changes occurring in maximal cross-correlations and latencies of AD mice when compared to age-matched WT mice. Warm and cold colors indicate increase and decrease, respectively. The color intensity reflects the level of statistical significance.

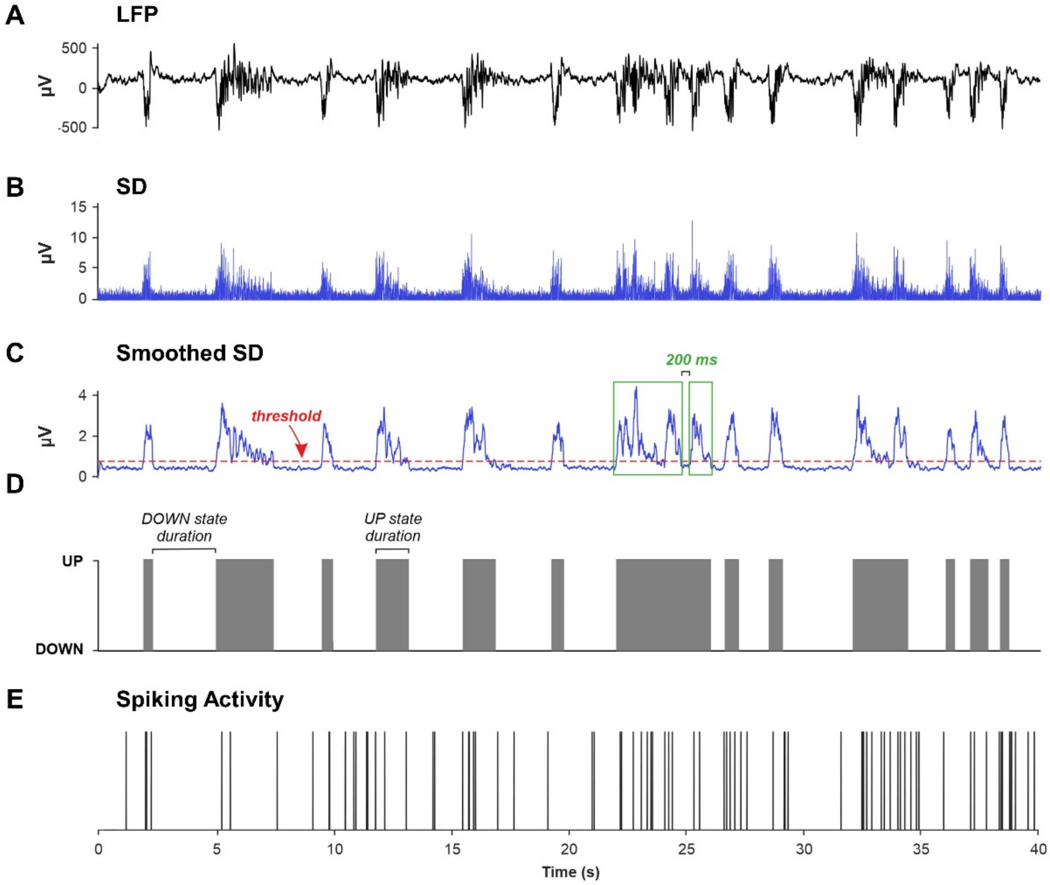

Supplementary Figure S11. Detections of UP- and DOWN-states and spiking activity. (A) Representative LFP trace employed for UP- and DOWN-state detection; (B) standard deviation (SD) of the LFP trace shown in (A), computed adopting sliding windows of 4 samples; (C) smoothed SD signal obtained with moving-average filter; the red dotted line indicates the corresponding threshold, while the green rectangles highlight two UP-states separated by less than half a second, merged together as a single UP-state; (D) classification of UP- and DOWN-states as events, respectively, above and below threshold; (E) spiking activity was detected as described in Supplementary Materials.

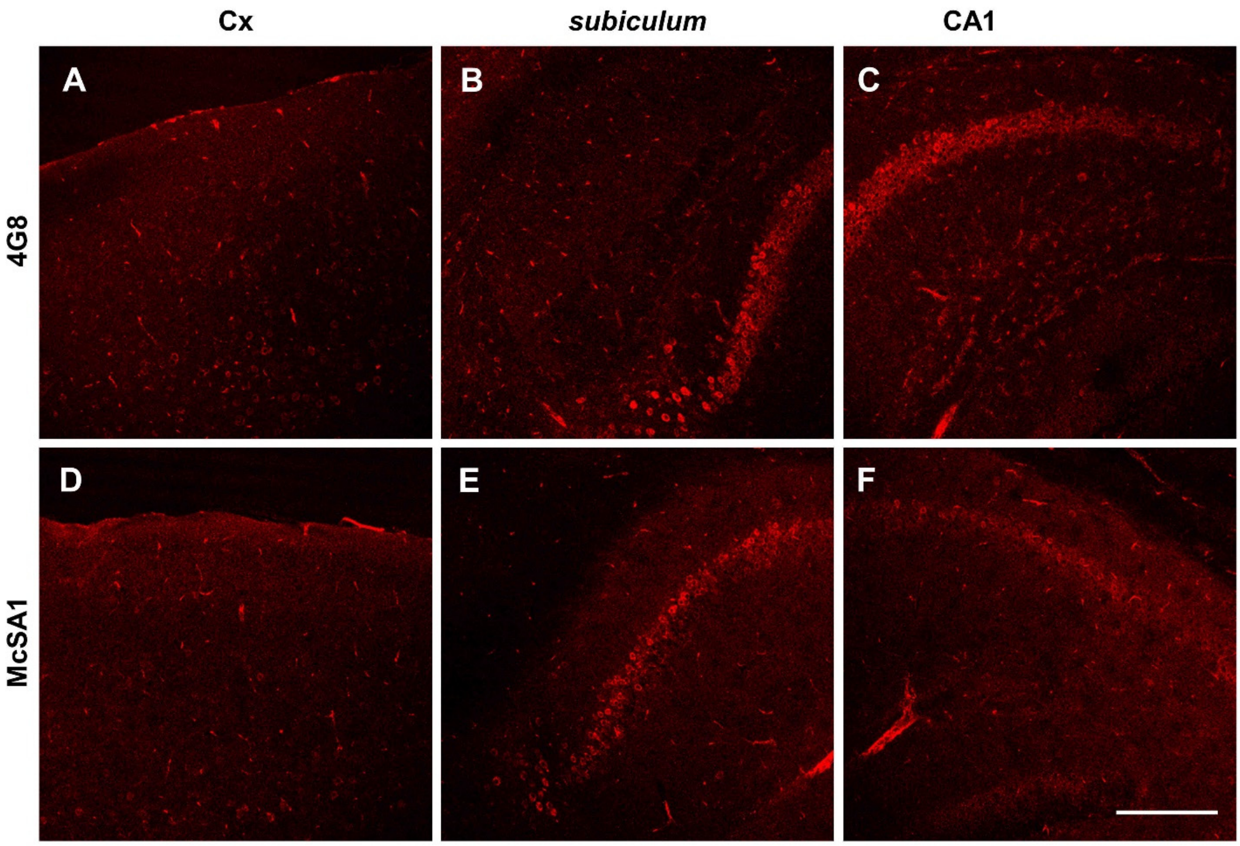

Supplementary Figure S12. Confocal images of sagittal slices of cortex (Cx, A, D), *subiculum* (B, E) and CA1 (C, F), stained with the anti-Aβ antibody 4G8 (top) or McSA1 (bottom) in 1-month-old AD mice (20x, scale bar, 200 µm). Both the antibodies detect intra-neuronal Aβ; the higher level of staining with 4G8 is likely due to its lesser specificity, detecting also full-length APP and its cleavage products.

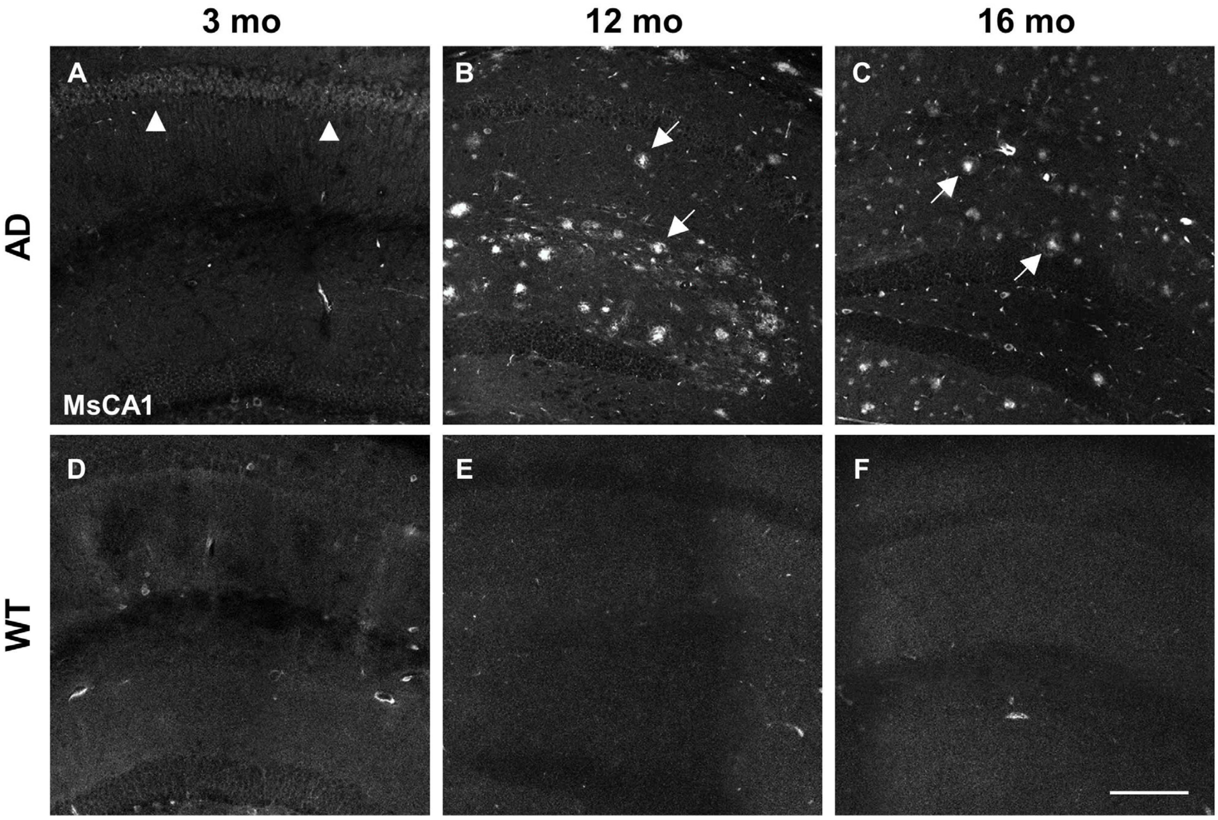

Supplementary Figure S13. Confocal images of cortical coronal slices from 3-, 12- and 16-month-old AD (A-C) and WT (D-F) mice stained with the anti-Aβ antibody McSA1 (20x, scale bar, 200 µm). Images from WT mice (D-F) were acquired at increased brightness/contrast. Arrowheads in panel A indicate intraneuronal Aβ, arrows in panels B, C extracellular plaques.

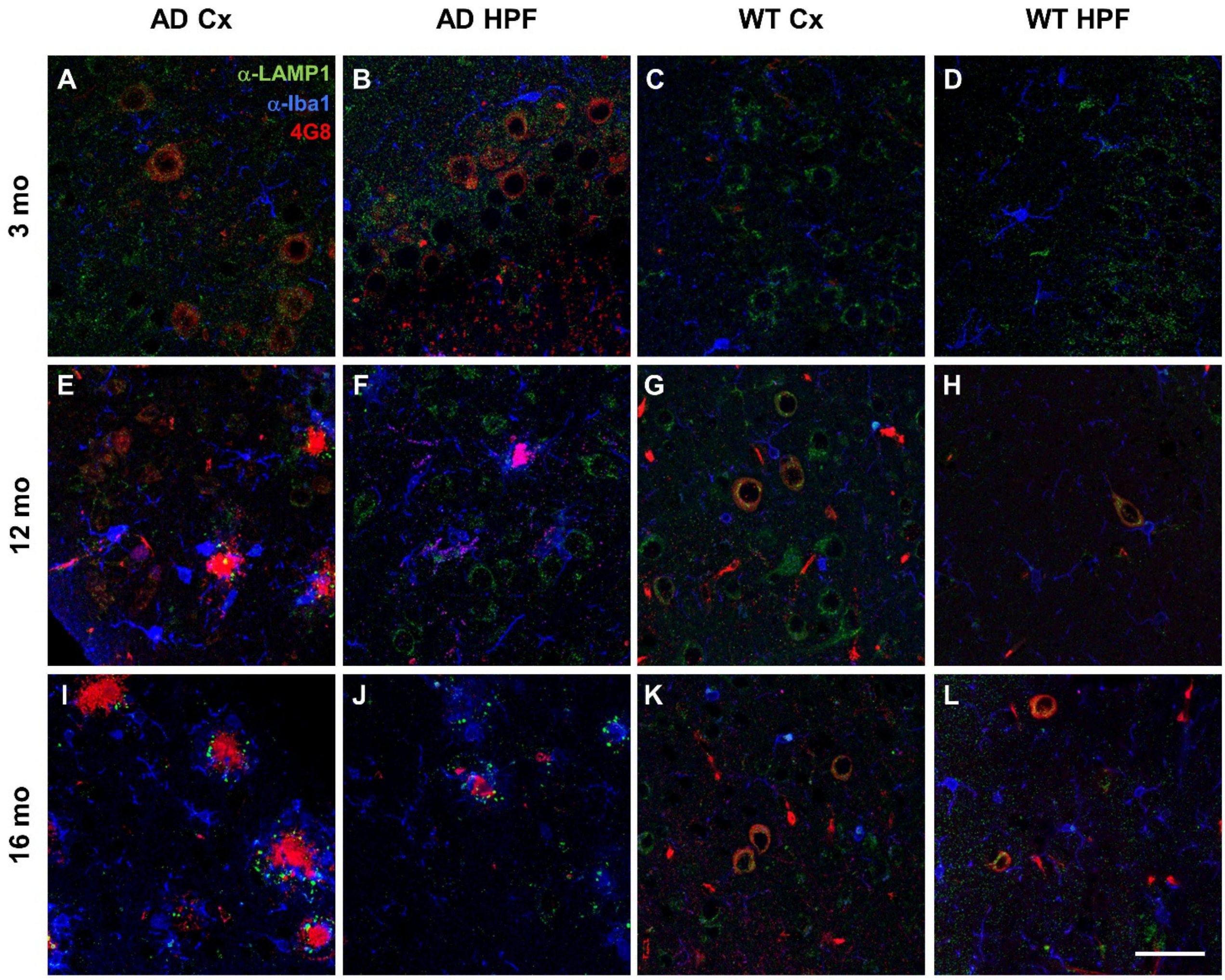

Supplementary Figure S14. Progress of neuroinflammation in AD mice. Confocal images of coronal slices of cortex (Cx) and hippocampal formation (HPF) from 3-,12-, and 16-months-old AD and WT mice. Immunostaining for Aβ (4G8, red) shows plaques surrounded by activated microglia (Iba1, blue) and aggregates of Lamp1 (green), indicative of dystrophic neurons (8); 100x, scale bar 40 μm. Images from WT mice were acquired at increased brightness/contrast to possibly highlight microgliosis and dystrophic neurons as found in 12- (E, F) and 16-month-old AD mice (I, J).
